# Ankle stiffness assessment in individuals with chronic ankle instability in dual-task single leg stance on an unstable surface

**DOI:** 10.1080/07853890.2021.1896440

**Published:** 2021-09-28

**Authors:** Nuno Dias, Hugo Estupendo, Isabel Valente, Luis Carmo, Andreia S. P. Sousa

**Affiliations:** ^a^Center for Rehabilitation Research, School of Health, Polytechnic of Porto, Porto, Portugal

## Abstract

**Introduction:**

The ankle sprain is one of the most prevalent traumatic injuries in sport, associated with a significant risk of developing Chronic Ankle Instability (CAI) [1,2]. This condition involves postural control deregulation expressed in feedback and feedforward mechanisms and stiffness as a consequence of incorrect or ineffective sensorial input that compromise the ankle joint stability [3]. This study aims to analyse the functional stiffness and postural stability in single leg stance on an unstable surface with dual tasking subjects with CAI.

**Material and methods:**

A cross sectional study was performed with a sample of 28 athletes of modalities of increased risk for ankle sprain. Participants were divided into two groups according to the presence (*n* = 14 (11 with mechanical ankle instability; 8 with CAI in the non-dominant limb), age 22.0 ± 2.225 years) or absence of unilateral CAI (*n* = 14, age 22.5 ± 1.75 years) identified through the Ankle Instability Instrument and the Foot and Ankle Outcome Score. The ground reaction forces, and centre of pressure (CoP) were assessed during dominant and non-dominant single leg stance during 30 sec on an unstable surface while the participants performed the Stroop test. The measures of CoP displacement, root mean square (RMS), standard deviation, velocity and area and functional stiffness were assessed through the data obtained by a force plate. Functional stiffness was assessed through the relation between the moment of force and the angular position of the ankle according to the method proposed by Winter [4]. This study was approved by the School of Health’s Ethical Committee and was performed in a research centre.

**Results:**

The CAI group showed an increase in the functional stiffness for mediolateral direction in the lesioned limb when this limb was the dominant one (*U* = 1.000; *p =* .001) but also when this limb was the non dominant one (*U* = 15.000; *p =* .033) and in the contralesional dominant limb (*U* = 7.000; *p =* .004). An increase in the RMS in the anteroposterior direction was observed in the lesioned limb (*U* = 13.000; *p = .*017), and a decrease in the CoP displacement in the non lesioned limb (*U* = 25.000; *p = .*034), in comparison to the control group but only when these limbs were dominant.

**Discussion and conclusion:**

Subjects with CAI seem to present increased bilateral functional stiffness as a possible strategy to increase postural stability in single leg stance, however stiffness assessments based on kinematics should be performed to confirm these findings. Rehabilitation should consider strategies to promote more efficient bilateral postural control mechanisms in subjects with chronic ankle instability.
